# Polyamines as Gatekeepers of Virus Replication and Central Nervous System Homeostasis

**DOI:** 10.3390/pathogens15040422

**Published:** 2026-04-14

**Authors:** Samantha P. Stacey, Bryan C. Mounce

**Affiliations:** 1 Department of Microbiology and Immunology, Loyola University Chicago, Maywood, IL 60153, USA; 2 Infectious Disease and Immunology Research Institute, Loyola University Chicago, Maywood, IL 60153, USA

**Keywords:** polyamines, metabolism, neurotropic viruses, central nervous system

## Abstract

Polyamines are small, positively charged molecules essential for fundamental cellular processes, including transcription, translation, and membrane fluidity. In the central nervous system (CNS), these molecules serve as homeostatic gatekeepers by modulating neuroreceptors like NMDA and supporting autophagic clearance. While basal polyamine levels are necessary for proper neuronal differentiation and memory formation, their dysregulation is a hallmark of neurodegenerative pathologies such as Alzheimer’s and Parkinson’s diseases. Neurotropic viruses, including poliovirus, Zika virus, and human cytomegalovirus are significant human pathogens that rely on cellular metabolites for their replication, including polyamines. These pathogens exploit polyamines at multiple stages of their life cycles, relying on them for virion stability, cellular attachment, and the stimulation of viral enzyme activity. Notably, diverse viral families share this dependence, making polyamine biosynthesis a prime target for broad-spectrum antiviral therapies. This review covers the current understanding of polyamine metabolism in virus infection and CNS health and disease, as well as considering antiviral therapies targeting host polyamines to limit neurotropic virus infection.

## 1. Polyamines in Cellular Biology

**Figure 1:**
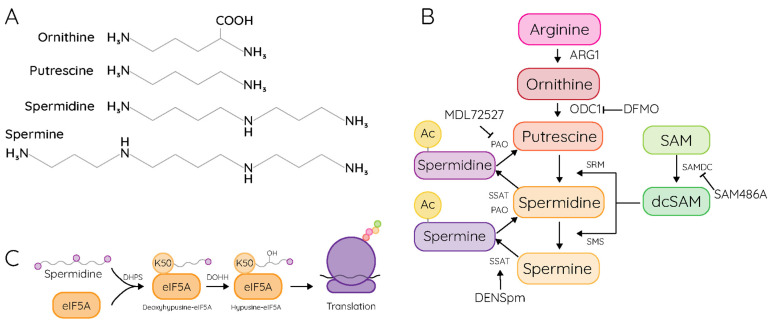
Polyamine structure, synthesis, and function in cellular translation. (**A**) The structures of the polyamines found in the human body and their precursor molecule, ornithine. (**B**) The polyamine biosynthetic pathway, the enzymes involved in their formation, and inhibitory or activating molecules. (**C**) The hypusination pathway, which supports cellular translation.

Polyamines are small, polybasic molecules, comprising short carbon chains and primary or secondary amino groups. At physiological pH, these polyamines are positively charged, and this charge facilitates their myriad functions within the cell and in an organism. Putrescine is the smallest of the polyamines, consisting of a four-carbon chain with terminal amino groups (Figure 1A). Spermidine and spermine have three and four amino groups, respectively. Additional natural polyamines include cadaverine and norspermidine, which are synthesized by some microorganisms and have specialized functions [1,2]. Polyamines are found abundantly in nearly all cells, at concentrations in the millimolar range [3]. However, polyamines can be toxic to some microorganisms, including *Staphylococcus aureus* [4,5]; thus, organisms have evolved distinct mechanisms of using, or avoiding, polyamines.

### 1.1. Polyamine Synthesis and Regulation

Eukaryotic cells synthesize polyamines in a stepwise manner from an arginine precursor derived from the urea cycle (Figure 1B). Arginine is converted to ornithine by arginase (ARG1), which releases urea for excretion. Ornithine is converted into the first polyamine, putrescine, through the action of ornithine decarboxylase 1 (ODC1), the gatekeeping step in polyamine synthesis. Conversion to putrescine is irreversible, and ODC1 activity mediates polyamine synthesis. ODC1 turns over rapidly within cells, with a half-life of approximately 10 min [6], and the cell expends significant energy transcribing and translating ODC1 [7]. ODC1 contains an internal ribosome entry site (IRES) to mediate its translation throughout the cell cycle [8]. ODC1 acts as a dimer and its activity is regulated by ODC1 antizyme 1 (OAZ1) [9,10], which has a similar structure to ODC1 but lacks a catalytic site [11,12]. OAZ1 binds to ODC1 and acts as a dominant negative to reduce ODC1 activity. OAZ1 itself is inhibited by antizyme inhibitor (AZIN), which binds OAZ1 to preclude its binding to ODC1 [11,13]. Each of these proteins is regulated transcriptionally and translationally by polyamine levels in the cell [14,15,16].

Once synthesized by ODC1, putrescine is rapidly converted into spermidine by spermidine synthase (SRM), and spermidine is subsequently converted to spermine by spermine synthase (SMS). Each of these reactions requires S-adenosylmethionine and the action of S-adenosylmethionine decarboxylase (SAMDC), which regulates these conversion steps. Distinct cell populations have different relative levels of each of these polyamines [17,18], which may reflect their specialized functions within the cell. The polyamines can be interconverted by the action of a polyamine acetyltransferase (spermidine-spermine acetyltransferase 1 [SAT1]) and a polyamine oxidase (PAOX), through which spermine can be converted to spermidine and, subsequently, putrescine. Acetylated polyamines can also be exported [19] or degraded in peroxisomes [20]. Thus, polyamines are heavily regulated by the cell, and the cell expends significant energy to maintain homeostatic polyamine levels.

### 1.2. Polyamine Functions

A key function of polyamines in eukaryotic cells is in translation. The aminobutyl group of the polyamine spermidine is conjugated to lysine 50 of eukaryotic initiation factor 5A (eIF5A) to form the modified amino acid deoxyhypusine (Figure 1C), catalyzed by the enzyme deoxyhypusine synthase (DHPS). Deoxyhypusine-eIF5A is subsequently hydroxylated by deoxyhypusine hydroxylase (DOHH), which generates the modified amino acid hypusine. Hypusinated eIF5A (eIF5A-hyp) is key to translation of a subset of cellular proteins [21]. The current model holds that eIF5A-hyp enhances translation of “hard-to-translate” proteins, especially those with diproline motifs [22]. eIF5A-hyp enhances peptidyl transferase activity and, as such, facilitates translational elongation [23]. Additionally, eIF5A-hyp may aid in translational termination, but this process is not entirely understood [21]. In bacteria, the closest analog to eIF5A is EF-P, but it is not modified by hypusination [24]. Polyamines still function in bacteria to facilitate translation, including by binding nucleic acids, including tRNAs, facilitating the formation of the initiation complex, and enhancing translational efficiency [25].

**Figure 2:**
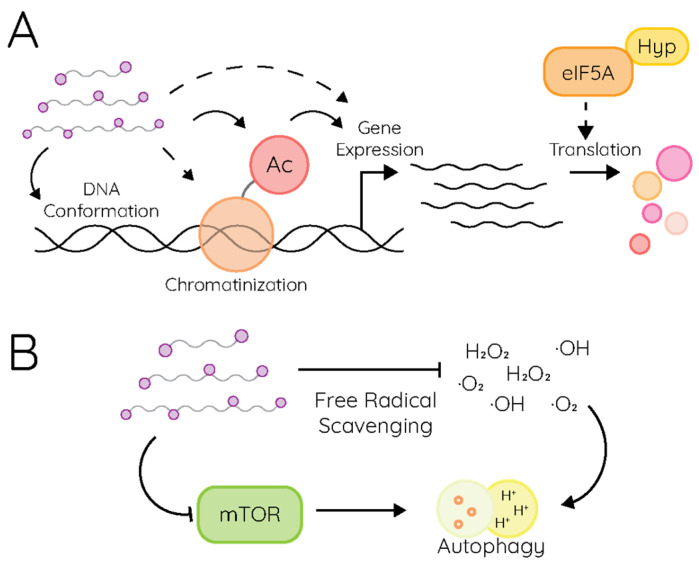
Polyamines in cellular processes. (**A**) The functions of polyamines in cellular DNA function, transcription, and translation. (**B**) Polyamines in a cellular signaling pathway, which mediates autophagy through mTOR and free radical scavenging.

The cell’s energy expenditure on polyamine synthesis and regulation underscores the importance of these molecules in cell homeostasis. As mentioned, polyamines are positively charged, and as such, they interact with negatively charged molecules, like nucleotides [26,27,28]. Polyamines can mediate DNA and RNA structure and, thereby, their function (Figure 2A). tRNA molecules bind to polyamines, and some tRNAs cocrystalize with polyamines [29,30,31]. mRNA structure and stability is also mediated by polyamines. DNA and histones can bind to polyamines and mediate chromatinization of the DNA and transcription of RNA [32,33]. The “polyamine modulon”—or polyamine-dependent transcriptional signature—has been described for some bacteria and includes genes involved in growth and replication [25]. Polyamines also bind to phospholipid headgroups and reduce membrane fluidity by limiting lipid movement within membranes [34,35]. Other functions of polyamines include participation in signal transduction pathways like autophagy signaling (Figure 2B) [36,37], protecting cells from oxidative stress by acting as free radical scavengers [38], and regulating enzyme activity [18,39].

## 2. Polyamines in the Nervous System

In the brain, polyamines are present in specific areas at high concentrations, as studied in both the human and rat brain [40,41,42]. For example, in whole rat brain homogenates, spermidine and spermine are enriched, whereas putrescine is present but detected at significantly lower concentrations. More specifically, putrescine is unevenly distributed in the rat brain [43]. Across several mammalian species, however, spermidine is typically present in the highest concentrations [7]. In white matter, specifically, there is a greater spermidine-to-spermine ratio compared to gray matter [43,44], highlighting differential regulation of polyamine species in the brain. The precise functional effects of these differences in polyamine abundance remains to be fully understood. Further, whether polyamines within these tissues support viral infection remains to be explored.

**Figure 3:**
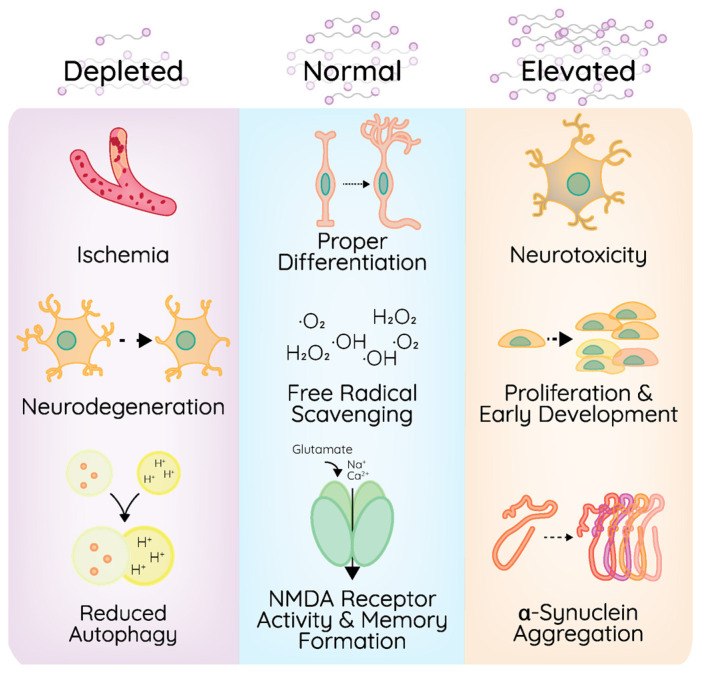
Polyamines in the central nervous system. Polyamine levels dynamically regulate critical processes in the central nervous system. Polyamines are depleted upon ischemia and neurodegeneration, and depleted polyamine levels reduce autophagic flux. Elevated polyamines result in neurotoxicity and α-synuclein aggregation but are also critical for proliferation and early development. Basal polyamine levels promote proper neuronal differentiation, free radical scavenging, and NMDA receptor signaling for memory formation.

Polyamines dynamically support a variety of functions within the brain (Figure 3). They act as antioxidants and aid in the biosynthesis of other antioxidants, which are implicated in protection from neurodegenerative diseases [45,46]. However, excessive amounts of polyamines in the brain can induce neurodegeneration and become neurotoxic in mammals [47,48]. Polyamines play a direct role in stress regulation, with implications for managing brain injury and the elevated levels of free radicals that are generated in the brain after injury. The polyamine stress response (PSR) in the brain becomes activated after stress stimuli or injury, and in these circumstances, the brain experiences a temporary increase in polyamine metabolism to adapt to stress [49]. Such phenotypes have been described during traumatic brain injuries and cerebral ischemia, specifically through elevated putrescine levels. Levels of spermine and spermidine may remain stable for a period but decrease under prolonged stress [50,51]. The PSR is developmentally regulated and is mostly active in mature brains [52]. This response must be temporary, as altered concentrations of polyamines can have dramatic effects on the nervous system, such as increased oxidative stress and DNA damage due to reduced antioxidant capacity [53]. Polyamine dysregulation, therefore, can lead to cell dysregulation and neurodegenerative disorders.

The process of aging is accompanied by changes in polyamine levels and their distribution. Studies have shown that polyamine levels are higher in infant brains compared to adult brains [40]. Age-related polyamine dysfunction has been reported in detail in mice, showing that the aging process leads to complex changes in the levels of each polyamine in different parts of the brain [54]. In mice, a polyamine agonist that can bind to NMDA inhibits the development of fear memories [55]. Therefore, polyamine levels may mediate memory formation through NMDA memory. Polyamine levels decrease with age, which may contribute to age-related memory loss. Polyamines, specifically spermidine, are important for autophagic clearance of proteins and cell debris, which have been linked to brain aging and neurodegenerative disorders, suggesting a connection between spermidine, autophagy regulation, and NMDA-mediated signaling. Interestingly, spermidine supplementation can improve memory in flies, potentially through induction of autophagy [56].

NMDA receptors contain a polyamine binding site, providing a potential molecular mechanism by which polyamines may mediate memory formation [57,58]. Binding of polyamines to the receptor causes activation, which promotes neurotransmission. However, excessive activation of these receptors leads to excitotoxicity, which can damage neural cells and harm memory recall [59,60]. Data suggest that the levels of polyamines in the brain are tightly controlled for proper memory formation through the NMDA receptor. Given these phenotypes, regulation of polyamine levels in the brain is critical for maintaining cellular functions, as elevated or depleted polyamines can negatively impact cellular functions.

Polyamines have been linked to several neurodegenerative disorders, perhaps best exemplified by the study of polyamines in Alzheimer’s disease. Patients with Alzheimer’s disease have elevated levels of spermidine and decreased levels of putrescine compared to healthy individuals [61]. Polyamine inhibition, either by drugs like difluoromethylornithine (DFMO), an ODC1 inhibitor, or agonists such as arcaine, can improve memory in Alzheimer’s disease mouse models, indicating the importance of polyamine regulation in the brain in the context of Alzheimer’s disease [62]. It is also known that polyamine dysregulation contributes to the development of Alzheimer’s disease by increasing cell death and aldehyde concentrations in the brain [63].

Similarly, polyamine dysregulation has been noted in Parkinson’s disease, as indicated by an abnormal decrease in spermine and an increase in spermidine and acetylated spermidine [64]. Polyamines have been described to advance the aggregation of alpha-synuclein, a common marker of Parkinson’s, prion, Alzheimer’s, and Huntington’s diseases [65,66,67,68]. Additionally, polyamines are dysregulated in the blood of individuals with treatment-resistant depression [69]. While polyamines may contribute to the development of Huntington’s disease, an injection of spermine was shown to improve memory and task completion in Huntington’s disease mouse models, paradoxically [70], again highlighting the importance of polyamine homeostasis for neurological function. Polyamines also impact the brain through cell protection and regeneration, promoting the proliferation, differentiation, and function of neural cells [56,71,72]. Polyamines’ roles in these neurodegenerative diseases, as well as neuroprotective mechanisms, are complex, however, and molecular mechanisms connecting polyamines to specific pathologies remain elusive. Given the pathological implications of elevated or depressed polyamine levels, careful regulation of these molecules is key for maintaining cellular functions in the central nervous system.

Viral infection of the CNS has been putatively linked to neurodegenerative disorders, like Alzheimer’s and Parkinson’s diseases or dementia. The “viral hypothesis” of neurodegeneration has gained traction following significant epidemiological studies that demonstrated that severe viral exposures, such as encephalitis, are associated with a significantly increased risk of developing Alzheimer’s and Parkinson’s diseases decades later [73,74,75,76]. Neurotropic viruses like HSV-1 and SARS-CoV-2 may drive these pathologies by compromising barriers, activating immune cells, and inducing protein misfolding and accumulation as part of an aberrant immune response. Importantly, polyamines may play a dual role in this landscape, both as proviral factors and as mediators of neurodegeneration.

## 3. Viruses of the Central Nervous System

Diverse viruses travel to and infect the human brain (Table 1). The viruses that infiltrate the brain or central nervous system are categorized as neurotropic, neuroinvasive, or neurovirulent. Viruses that infect the brain and either the peripheral or central nervous system are categorized as neuroinvasive. Viruses that cause disease in the brain or nervous system are neurovirulent. Neurotropic viruses infect and produce viral progeny in brain and nervous system cells [77,78]. Each category of virus may also respond to treatments differently within the nervous system. The blood–brain barrier serves as an obstacle for viruses to infiltrate the brain and nervous system due to its low permeability. However, some viruses can pass through epithelial layers or travel through local peripheral nerves to establish infection in the brain and nervous system. This may occur when a virus directly damages the blood–brain barrier (BBB) or if the virus can biochemically disrupt the tight junctions between the epithelial cells of the BBB [79]. The blood cerebrospinal fluid barrier (BCSFB) also provides another barrier to invading viruses [79,80]. An example of this is inflammation caused by Toll-like receptor 3 (TLR3), which can disrupt BBB epithelial cells, allowing the virus to gain access to the cells of the nervous system [79,81]. Viruses can also invade the CNS through *trans* neural invasion and retrograde axonal transport [79]. This section will cover a small subset of the neurotropic, neuroinvasive, and neurovirulent properties of some of the most well-known viruses that can infiltrate the brain and nervous systems. We also highlight how previous studies have identified a potential role for polyamines in these viruses’ replication, which may have implications for treating these infections.

### 3.1. Poliovirus

Poliovirus is a picornavirus that causes poliomyelitis. Poliovirus is not exclusively neurotropic and is primarily an enteric virus that occasionally causes severe disease in the central nervous system. In the intestine, the virus invades nearby lymph nodes and then spreads through the lymphatic system. Poliovirus then spreads through the bloodstream to infect new tissues. This viremia shuttles the virus toward the brain. Poliovirus has also been shown to alter the permeability of the BBB and travel to the CNS through peripheral nerves by retrograde transport [82,83,84,85]. However, this phenomenon is quite rare [86]. Poliovirus infection specificity is determined by the poliovirus receptor (PVR) [85], and poliovirus can become neurotropic after motor neuron infection, specifically, which possesses the poliovirus receptor [86,87,88]. Poliovirus replication damages and kills these neurons, which can lead to typical poliomyelitis symptoms [85]. Poliovirus is therefore a neurotropic, neuroinvasive, and neurovirulent virus. Previous work has shown that poliovirus relies on polyamines for replication [89]; however, the mechanism(s) by which polyamines function in poliovirus infection remain to be understood, and the roles of polyamines in poliovirus infection of the central nervous system is unexplored.

### 3.2. Rabies Virus

Rabies virus, a single-stranded negative-sense RNA lyssavirus from the Rhabdoviridae family, targets motor neurons by traveling across peripheral motor nerves and neuromuscular junctions and spindles [90]. Rabies virus then travels through the nervous system through neurons and eventually makes its way to the salivary glands to transmit to additional hosts [91]. This process is facilitated by nicotinic acetylcholine receptors (nAChR), which are only present in muscle cells, which the virus binds to initiate entry into the host cell [92,93]. After exiting these muscle cells, the virus then enters nerve synapses located in the surrounding muscle tissue. This process allows the virus to access the CNS and disperse using retrograde axonal transport through motor neurons [92,94,95,96]. Rabies virus demonstrates the ability to proliferate extensively in neurons, the main cause of neurological symptoms [97]. The symptoms start to appear after the virus has extensively replicated and invaded the CNS, at which point treatment is ineffective and almost exclusively leads to host death. As such, rabies virus has neuroinvasive, neurotropic, and neurovirulent qualities. However, the disease does not seem to cause complete cell death or neuronal loss but rather neuronal dysfunction [98]. Polyamines stimulate rabies virus infection in vitro, specifically using primary cortical neurons [89].

### 3.3. Arboviruses: Zika Virus and West Nile Virus

Zika virus (ZIKV) is a positive-sense neurovirulent RNA virus that causes disease in infants and adults. During pregnancy, the fetus can develop microcephaly and other disorders if the mother is infected with ZIKV and the virus transmits to the fetus. ZIKV infection may also cause the fetus to develop congenital Zika syndrome (CZS), which impairs proper development and causes abnormalities in various body systems of the fetus [99]. Adults can develop Guillain-Barré syndrome or encephalitis due to the neurotropic qualities of ZIKV [100,101,102,103,104]. ZIKV is a flavivirus transmitted by *Aedes* mosquitoes or via sexual contact, though it can be transmitted from mother to child during birth [105,106,107]. The fetal nervous system has many barriers to protect the developing fetus, including the placental and blood-brain barriers. However, if the virus gains access to the fetal brain, ZIKV specifically targets and replicates in neuronal progenitor cells, though it does not seem to target developing neurons [108,109]. This broad cell tropism mediates neuroinvasion and gives the virus access to many parts of the body in both adults and fetuses.

Importantly, ZIKV can bypass the BBB and the placental barrier [110]. Recent studies have demonstrated that ZIKV does not alter the permeability of the BBB, which is often seen in other CNS infections [111,112]. Instead, ZIKV utilizes the process of transcytosis. Once ZIKV infects the epithelial cells that make up the BBB, viral particles exit the basal side of the cells, allowing the virus to infect nearby cells [110]. ZIKV can invade cells through clatherin-mediated endocytosis and the interactions between the virion glycoproteins with cell receptors, including AXL, TYRO3, and others, which are widely expressed in the fetal brain [113,114].

Some of the symptoms that occur during ZIKV infection may be due to excessive inflammation. Research suggests that ZIKV can increase the presence of CD8^+^ T cells and inflammatory molecules during infection, which can cause neurological complications such as encephalitis [115,116,117]. ZIKV also causes neuronal progenitor cells in the cortex to decrease in size and limits proliferation and development of these cells, which contributes to microcephaly [109,118,119,120]. Death of these neural progenitor cells after infection by ZIKV also plays a role in the development of microcephaly [107,121]. In vitro studies also demonstrated that ZIKV has the potential to infect cortical astrocytes, microglia, and oligodendrocytes, and neuroepithelial stem cells in the fetal brain, which inhibits the proper development of the brain during pregnancy [114,120,122,123,124].

Similar to ZIKV, West Nile virus (WNV) is a mosquito-borne flavivirus. Most people infected with WNV do not experience symptoms, and WNV is rapidly cleared. Those who do experience symptoms are generally the elderly and immunocompromised, who are at risk of neurological sequelae, such as encephalitis and meningitis [125]. WNV can invade the CNS by altering the permeability of the BBB, as well as infecting and traveling within immune cells to the brain [126,127,128,129]. WNV, however, must reach the BBB from the site of a mosquito bite, which it transits by first infecting Langerhans cells in the skin. The virus then travels to and infects regional lymph nodes, leading to viremia [130,131]. The infected blood then encounters the BBB. The presence of WNV in the brain and CNS triggers increased levels of leukocyte recruitment and inflammation, which negatively impact the function of the BBB, allowing the virus access to the CNS. WNV can also use a “Trojan horse” mechanism, infecting immune cells and traveling in them through the BBB to infiltrate the CNS [132,133]. Finally, WNV enters the CNS through vascular endothelial cells, olfactory neurons, and a process known as axonal retrograde transport, the process of moving biological materials from axon terminals to the neuronal cell body [134,135]. WNV can infect neurons, astrocytes, and glial cells [136,137,138]. In neurons, however, WNV infection causes cell death. For glial cells, WNV induces the production of cytokines. Together, these factors lead to severe disease [136,139,140].

Initial work on polyamines in arbovirus infection demonstrated that the molecules facilitate ZIKV infection at the stage of genome replication [141]. Varied modulators of the polyamine pathway, such as DFMO, limit ZIKV infection, and multiple strains of ZIKV are sensitive to these inhibitors as well [89]. No studies have been performed in vivo to test the function of polyamines in ZIKV pathogenesis, but in vitro assays have shown significant promise in reducing virus replication. Further work on arboviruses like WNV, dengue virus (DENV), and chikungunya virus (CHIKV), among others [89,141], has highlighted that these viruses rely on polyamines, including at the level of viral translation, which will be explored in the next section. This polyamine dependence was also observed in mosquito cells [141], suggesting that polyamines facilitate arbovirus replication in both the mammalian and insect host.

### 3.4. Human Cytomegalovirus

Human cytomegalovirus (HCMV), like ZIKV, targets and infects the fetal brain during pregnancy. HCMV is a common betaherpesvirus that infects a majority of the world’s population [142]. The pathogen remains latent in infected individuals for life, yet the virus often does not cause symptoms for those infected [143,144]. The lack of symptoms is due to a robust immune response that limits virus replication and damage to the host. However, HCMV can be dangerous for immunocompromised individuals and fetuses [143,145]. Mothers infected with HCMV can vertically pass the virus to their child during pregnancy, which has the potential to interfere with fetal development and cause birth defects. HCMV can lead to conditions such as microcephaly and cerebral palsy or may cause epilepsy or frequent seizures [146,147,148,149]. HCMV infects neuronal progenitor cells (NPCs) like ZIKV at every gestational age [150]. NPCs infected with HCMV cannot differentiate properly and have elevated rates of apoptosis due to infection [151]. In mice, murine cytomegalovirus, or mCMV, infects and replicates in mature neurons and glial cells. mCMV also infects other cell types in the adult mouse brain, including radial glia, which help with neuronal migration [152]. Other in vitro studies found that HCMV inhibits neural stem cell development and neuronal migration, which are critical for proper brain function [153,154]. HCMV can infect brain endothelial cells, which allows the virus to cross the BBB and infect other brain cells [155,156]. HCMV, therefore, can infect and replicate in various brain cells, which can result in disease due to development inhibition and inflammation, making the virus neuroinvasive, neurotropic, and neurovirulent [157].

Polyamines in herpesvirus infection have been studied for decades and have highlighted unique ways that these viruses interact with cellular polyamines, as described more fully in the next section. Initial studies on herpesviruses focused on measuring levels of polyamines in infected cells and in infectious virions [158,159], demonstrating that herpesvirus infection induces polyamine metabolism and that these molecules are packaged in released infectious virions, and more recent work has demonstrated that polyamines facilitate viral translation and transcription [160,161] via mechanisms that share some commonalities with other, most distantly related viruses.

## 4. Polyamines in Virus Infection

Viruses are intracellular pathogens, and as such, they must utilize the host cell’s resources for replication. One of these resources is polyamines, which function in virus infection in similar ways to how they function within the eukaryotic cell [162,163,164]. Initial studies of polyamines in virus infection focused on measuring polyamine levels in infected cells, and in several instances, virus infection stimulated cellular polyamine synthesis [165]. This was perhaps best described for human cytomegalovirus (HCMV), which pushes cells into a pseudo-S phase, along with increasing polyamine levels within the cell [166]. Polyamines were also studied as structural components of viruses, and several viruses were found to package polyamines within their virions [158]. However, the precise functions of these polyamines were unclear at that time, and still today, several open questions remain about how polyamines function in virus infection. Interestingly, viruses of nearly all families tested to date exhibit dependence on polyamines for at least one step in the virus replication cycle, and understanding how polyamines function in the context of virus infection has significant potential for uncovering broad-spectrum antiviral therapies.

Initial investigations of purified virions identified several viruses that incorporate polyamines into their virions. Herpesviruses, including herpes simplex virus 1 (HSV-1), have both spermidine and spermine in the virion, with spermine primarily within the capsid and spermidine in the space between the lipid envelope and the capsid, called the tegument [158]. Vaccinia virus, a poxvirus, also incorporates spermidine and spermine [167]. In contrast, RNA viruses like rhinovirus or poliovirus did not have detectable levels of polyamines within the virion [168]. The precise function of polyamines within the virion have not been entirely understood; however, a leading hypothesis is that polyamines facilitate condensation of the viral nucleic acid, which mediates packaging of the negatively charged DNA into the small capsid space [169,170]. In addition, the concentration of polyamines within the capsid may mediate ejection of the viral DNA upon infection of a new host cell [171]. More recent reports have demonstrated that polyamines are components of bunyavirus particles [172], and these polyamines mediate virion stability and infectivity. This may be partly due to the polyamines functioning in lipid metabolism [173,174] and the genesis of the lipid envelope of the virus.

**Figure 4:**
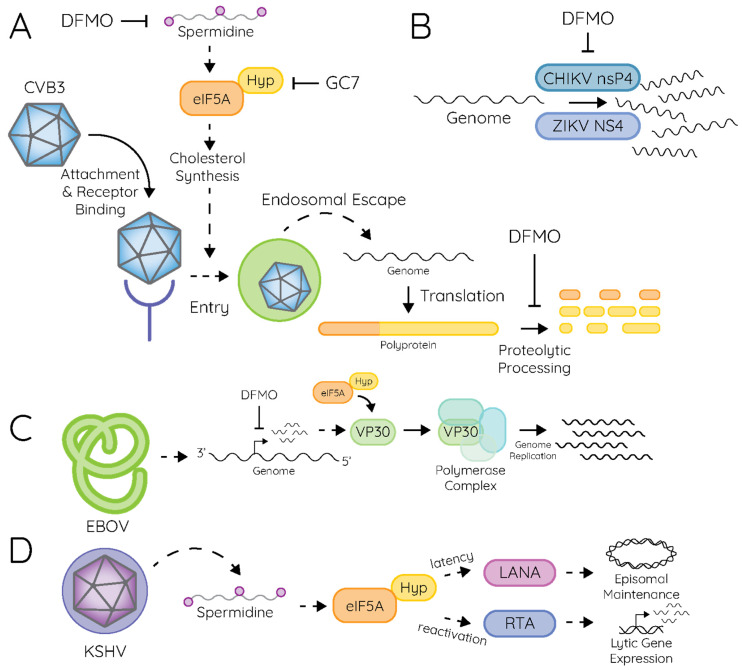
Polyamines in viral infection. (**A**) Polyamines mediate Coxsackievirus B3 (CVB3) infection at the stages of entry and proteolytic processing. (**B**) Polyamines facilitate viral polymerase activity for chikungunya virus (CHIKV) and Zika virus (ZIKV). (**C**) Ebolavirus (EBOV) and filovirus replication relies on polyamines for viral gene expression and the translation of VP30 for production of the viral replicase complex. (**D**) Kaposi’s sarcoma-associated herpesvirus (KSHV) modulates spermidine and hypusinated eIF5A levels to regulate latent genome maintenance through the translation of LANA and viral reactivation through the translation of RTA.

More recent work highlighted additional functions of polyamines in viral infection (Table 2), including for viruses of the central nervous system. For example, polyamines mediate attachment of viruses to cells, as well as the downstream entry events (Figure 4A). Specifically, in the enterovirus model system Coxsackievirus B3 (CVB3), polyamines mediate attachment of the virus to susceptible cells, as depleting polyamines via difluoromethylornithine (DFMO), an inhibitor of ODC1, reduced CVB3’s ability to attach [175]. Interestingly, incubating virus directly with polyamines rescued this defect [176], suggesting a direct function of polyamines in mediating virus attachment. Precisely how polyamines may directly mediate attachment remains to be fully understood; however, several intriguing hypotheses could explain these observations. First, polyamines may garner a net-positive charge on the virion, mediating interaction of positively charged virions with negatively charged cell surface molecules like heparan sulfates. Additionally, polyamines may directly mediate the ability of the virus to engage with its cellular receptor (the Coxsackievirus and adenovirus receptor [CAR] in the case of CVB3).

In addition to the role of polyamines in directly mediating virus attachment, other work highlighted that polyamines mediate cellular functions to promote viral attachment and entry. In polyamine-depleted cells, supplying cells with exogenous polyamines rescued viral attachment [175,179]; however, the rescue required an extended incubation time of approximately four hours, suggesting a role for a cellular process [174]. It was found that cellular cholesterol, an essential molecule for CVB3 attachment and entry [193], depends on cellular polyamines. Specifically, polyamines mediate the translation of sterol response element binding protein 2 (SREBP2), which is a key transcription factor for a host of cholesterol synthesis genes [174]. Thus, depleting polyamines reduced viral attachment through the depletion of cellular cholesterol. Additional polyamine-modulated cellular pathways likely function in viral attachment and entry, as well as other steps in viral replication, but have yet to be discovered.

As aliphatic compounds, polyamines can act as a countercharge to anions, which has been explored in cells with relation to how polyamines interact with nucleic acids or lipids. Polyamines also putatively bind to proteins and mediate their functions, including viral proteins. The best-described mechanism by which polyamines mediate viral enzyme function is with viral polymerases (Figure 4B), as early investigations showed that polyamines enhance transcription of bacteriophage genomes [169,194]. In fact, polyamines are a key component of in vitro transcription reactions because of their ability to stimulate polymerase activity. Further work has shown that polymerases from diverse viral families are sensitive to polyamines, including alphaviruses, filoviruses, enteroviruses, herpesviruses, and flaviviruses, among others [141,195,196]. These phenotypes are not limited to polymerases, however, as polyamines also stimulate protease activity in enteroviruses [180,181] and helicase activity in hepatitis C virus infection [197]. In fact, studies have shown that multiple hepatitis C virus enzymes are sensitive to polyamines [197], suggesting that the virus has evolved a reliance on polyamines for several steps in infection.

Work on alfalfa mosaic virus (AMV) showed that polyamines stimulate translation of the virus by 1.5- to 2-fold [198]; however, it was never uncovered precisely how polyamines may mediate this translation. We now know that polyamines mediate translation through eIF5A hypusination, which facilitates translation of a subset of cellular transcripts. In particular, translation of “hard-to-translate” regions of proteins is mediated by eIF5A hypusine, including diproline motifs [24], which may pose a challenge to the elongating polypeptide in the ribosomal exit tunnel. eIF5A hypusination has also been shown to facilitate translation of viral proteins, first in filoviruses (Figure 4C). In the Ebolavirus and Marburgvirus systems, VP30 specifically relied on polyamines and eIF5A hypusination for its protein accumulation, while other virus proteins did not [183,199]. Further, transcription of VP30 was not affected, specifically implicating translation in the polyamine-mediated regulation. More recent work on Kaposi’s sarcoma-associated herpesvirus (KSHV) also showed that hypusination facilitates the translation of the key viral proteins LANA and RTA (Figure 4D), and depletion of polyamines or hypusination limits viral translation and replication [160,161]. Interestingly, KSHV modulates the levels of polyamine synthetic genes, like ODC1, and hypusinated eIF5A, which mediates lytic and latent infection. Thus, the virus has evolved a relationship with polyamine metabolism and exploited it to regulate its lifecycle. Other viruses have been shown to manipulate polyamines, especially the herpesviruses and vaccinia virus [191,192]. Interestingly, some viruses, especially large aquatic DNA viruses like *Paramecium burcella* chlorella virus (PBCV) encode their own polyamine synthetic pathway [200,201,202,203].

Several of the above-mentioned viruses can infect the central nervous system, including the enteroviruses and herpesviruses, for example. The neurotropic nature of these viruses does not appear to connect to polyamines, as no specific studies have considered neuropathogenesis of these viruses in the context of a polyamine-depleted host. In fact, the roles of polyamines in viral pathogenesis remain inadequately studied in general. However, it is tantalizing to develop models that could connect specific cellular tropism to polyamine metabolism. For instance, given virus’ penchant for polyamines, they may specifically infect cells with sufficient polyamine resources, and, in contrast, cells may react to infection by restricting this pool. Such complex regulation of infection remains to be identified, and significant further investigation is required to tie tropism to polyamine metabolism.

## 5. Targeting Polyamines as an Antiviral Therapy

While polyamines are important for overall cellular function, they also promote viral infections, as discussed above. Therefore, depleting polyamines could potentially be an antiviral strategy for viruses, including those that infect the central nervous system. Previous studies showed that inhibition of polyamine biosynthesis by DFMO, an FDA-approved drug, can reduce viral titers, including neurotropic viruses like Zika virus, poliovirus, and rabies virus. DFMO functions by blocking cellular synthesis of polyamines, acting as a potent inhibitor of ornithine decarboxylase 1 (ODC1), the first committed step in polyamine synthesis. While the antiviral activity of this drug has not been studied in the context of the CNS, current research suggests that DFMO could be useful against neurotropic viruses because DFMO reduces virus replication in vitro [89]; however, further research is needed to confirm if DFMO would be effective against neural viruses, such as in neural cell culture or in the brain of an in vivo model. Prior in vivo work supports this potential: DFMO has been shown to reduce chikungunya virus (CHIKV) and CVB3 titers in mice [89], demonstrating that polyamine inhibitors such as DFMO may have therapeutic potential.

Polyamines can also be reduced through upregulation of the enzyme spermidine-spermine acetyltransferase 1 (SAT1), which is accomplished by treatment with diethylnorspermine (DENSpm), which was also shown to reduce viral titers [89,179,182]. In DENSpm treated cells, SAT1 is potently induced, which results in the depletion of spermidine and spermine and the accumulation of putrescine. SAT1 expression caused by type 1 interferon signaling also leads to lower polyamine concentrations in the cell. This mechanism has been shown to prevent Zika virus replication [141]. Thus, targeting polyamines may serve as a powerful therapy and could be a valuable tool for combating diverse viruses, including those that infect the CNS.

N,N′-Bis(2,3-butadienyl)-1,4-butanediamine dihydrochloride (MDL 72527) is a spermine analog and inhibitor of polyamine oxidase (PAO) [204,205]. Similar polyamine analogs, such as guazatine, have the same targets and similar effects as MDL 72527 [205]. However, there is limited evidence supporting the antiviral properties of these polyamine analogs. Similarly, a spermidine analog known as N1-guanyl-1,7-diamine-heptane (GC7) inhibits hypusination that is crucial to virus infection, as described above. Spermidine is used as a substrate to generate hypusinated eukaryotic translation initiation factor 5A (eIF5A), and GC7 blocks deoxyhypusine synthase (DHPS) by binding to the spermidine-binding site of this enzyme, which inhibits downstream hypusination of eIF5A [206,207,208]. This compound inhibits several viruses, such as vesicular stomatitis virus (VSV), ZIKV, CHIKV, and CVB3 [174,209].

While unexplored in the context of viral infection, polyamine-modulating therapeutics have been explored in the context of the central nervous system. Individuals with neurological diseases may see distinct effects from polyamine therapy [133,136], especially given the myriad roles of polyamines in the brain. Studying the role of polyamine biosynthesis in specific disease states can help us to understand more about the importance of this pathway and potential consequences from the use of polyamine inhibitors in humans. Several studies have shown that brain injury can lead to increased expression and activity of ODC1, leading to elevated levels of putrescine. The increased putrescine levels have been seen after the induction of epilepsy in mouse models. It was shown that treatment with DFMO could reduce the increased putrescine levels in these mice as well, but it is unclear whether this drug had any effect on recovery in these studies [210,211,212,213]. However, another study found that a DFMO pre-treatment, prior to electrical stimulation, had accelerated behavioral kindling, or that they developed increased seizure susceptibility at an accelerated rate [214]. This suggests that ODC1 may be both neuroprotective and neurodegenerative, depending on the disease context.

The dual role of ODC1 has also been seen in transgenic ODC1 overexpression mice. Researchers observed that these mice had an elevated seizure threshold but also diminished memory and spatial learning capabilities [215]. Delayed ODC1 and putrescine increases have also been observed for cerebral ischemia [216,217,218]. One study found that ODC1 was not the sole contributor to this increase in putrescine. Instead, they found that PAO activity also contributed and noticed that the drug MDL72527 could reduce elevated putrescine after an ischemic event. MDL72527 was also found to reduce brain edema in the cortex and ischemic brain injury volume in the cortex and subcortex [219].

Diseases directly resulting from mutations of genes involved in polyamine synthesis have been described. One example of such a “polyaminopathy” is Bachmann-Bupp syndrome (BABS) which is caused by mutations in ODC1 [220,221]. This mutated protein avoids degradation by the proteosome, which limits the cells’ ability to regulate its activity. Therefore, individuals with BABS often suffer from developmental delays and other neurological issues. DFMO, however, has been shown to help improve the symptoms of BABS patients [222,223]. Another similar genetic disease is the Synder-Robinson syndrome, caused by mutations in the SMS gene [224,225]. Mutations in polyamine genes may also play a role in autism spectrum disorder (ASD), particularly the gene SAT1 and potentially ODC1 and SMS, but research on this is limited [226,227,228]. The effect of drugs like DFMO in Snyder-Robinson Syndrome or ASD has not yet been explored, but preliminary evidence seen with BABS patients suggests they could treat similar syndromes. DFMO has also been FDA-approved as a treatment for *Trypanosoma brucei gambiense* encephalitis and pediatric high-risk neuroblastomas [229,230]. Therefore, polyamine inhibitors could be beneficial for treating certain conditions.

Beyond their potential as therapeutic targets through analogs and inhibitors, polyamines function as endogenous biomarkers that reflect the physiological state of the body, including during viral pathogenesis. Because polyamine metabolism is strictly regulated to maintain cellular homeostasis, significant deviation in their concentrations, either in systemic circulation or localized tissues, often signals underlying issues. In the context of the central nervous system, this is especially pronounced; for instance, patients with HIV exhibit significantly elevated levels of polyamines within both brain tissue and CSF [231]. This accumulation suggests that polyamines could serve as robust clinical markers not only for the presence of a viral infection but also for monitoring disease progression and the development of associated neurological complications, such as HIV-associated neurocognitive disorders (HAND).

Given this dependence of viruses on polyamines, targeting the polyamine biosynthetic pathway offers a compelling strategy for broad-spectrum antiviral intervention within the CNS. The use of inhibitors such as DFMO provides a blueprint for depleting the intracellular pools of polyamines that viruses require. Unlike traditional antivirals that target specific viral enzymes and thus remain susceptible to the rapid evolution of viral resistance, polyamine depletion targets a fundamental host requirement common to diverse viral families. Future therapeutic directions must therefore focus on the dual benefit of these interventions: halting viral expansion while simultaneously stabilizing the CNS polyamine “gatekeepers” to prevent neurotoxicity often associated with neurotropic virus infections. This integrated approach holds the potential to reshape our treatment of complex neurological viral diseases, moving toward therapies that protect both cellular homeostasis and genomic integrity. Given the difficulty in treating neurotropic virus infection, particularly with infections like rabies virus or herpes encephalitis, exploring novel antiviral strategies, such as polyamine depletion, offers significant potential.

## 6. Summary

Polyamines occupy a unique position at the intersection of cellular metabolism, neuronal physiology, and viral replication. Within the CNS, these metabolites contribute to diverse processes including regulation of ion channels, modulation of synaptic signaling, nucleic acid stability and function, and control of translation. At the same time, viruses rely on polyamines to support efficient replication, using them to maintain genome stability, translation, and enzymatic activity. The convergence of these roles highlights polyamine metabolism as an important host pathway that links fundamental aspects of CNS biology with the replication strategies of neurotropic viruses.

Despite growing recognition of this connection, many aspects of the relationship between polyamine biology and neurotropic virus infection remain poorly understood. Key questions remain regarding how polyamine metabolism is regulated within different neural cell types, how viral infection reshapes these metabolic networks during CNS infection, and whether changes in polyamine homeostasis contribute directly to neuropathogenesis. In addition, the CNS presents a unique metabolic environment, shaped by cellular specialization and the constraints of the blood–brain barrier, that may influence how viruses access and utilize polyamine pools. Addressing these questions will require integrated approaches that combine virology, neurobiology, and metabolic profiling to better define how polyamine dynamics influence viral infection in neural tissues.

A deeper understanding of polyamine-dependent processes in the CNS may also reveal new therapeutic opportunities. Pharmacologic modulation of polyamine synthesis or utilization has already shown antiviral potential in several viral systems, suggesting that host-directed targeting of this pathway could represent a broadly acting strategy against neurotropic viruses. However, given the essential roles of polyamines in neuronal function and CNS homeostasis, therapeutic approaches must balance antiviral efficacy with preservation of normal cellular physiology. Continued investigation into the mechanisms that govern polyamine metabolism during infection will be critical for defining how this pathway can be leveraged to combat neurotropic virus disease while maintaining the delicate functional balance of the central nervous system.

## Figures and Tables

**Table 1:** A subset of viruses of the central nervous system. Diverse viruses infect and cause disease in the central nervous system, with distinct mechanisms of infection and cell tropism.

Virus	Type	Mechanism of Infection	Cell Tropism
Poliovirus	+ssRNA	Retrograde axonal transport, hematogenous spread	Neurons (specifically motor neurons)
Rabies virus	-ssRNA	Retrograde axonal transport	Neurons
Zika virus	+ssRNA	Hematogenous spread, disruption of placental barrier	Neuronal progenitor cells, glial and microglial cells
West Nile virus	+ssRNA	Hematogenous spread, blood–brain barrier disruption, infected immune cells	Neurons, astrocytes, microglial cells
Human cytomegalovirus	dsDNA	Hematogenous spread	Neuronal progenitor cells, glial cells

**Table 2:** Summary of studies related to polyamines in human viruses. Polyamines play diverse roles in viral infection, facilitating replication at different stages and via distinct mechanisms.

Family	Virus	Type	Effects of Polyamine Depletion	Refs
Alphavirus	Chikungunya virus	+ssRNA	Decreased viral RNA polymerase activity, reduced titers	[89,141]
	Semliki Forest virus	+ssRNA	Decreased viral RNA polymerase activity	[177,178]
Coronavirus	MERS coronavirus	+ssRNA	Reduced titers	[89]
	SARS-CoV-2	+ssRNA	Reduced titers	[179]
Flavivirus	Japanese encephalitis virus	+ssRNA	Reduced titers	[89]
	Zika virus	+ssRNA	Decreased viral RNA polymerase activity, reduced titers	[89,141]
	Yellow fever virus	+ssRNA	Reduced titers	[89]
Enterovirus	Coxsackievirus B3	+ssRNA	Reduced titers, decreased viral attachment	[89,141,180,181]
	Enterovirus A71	+ssRNA	Reduced titers	[89]
Bunyavirus	Rift Valley fever virus	-ssRNA	Reduced titers, production of noninfectious virions	[89,182]
	La Crosse virus	-ssRNA	Reduced titers, production of noninfectious virions	[182]
Filovirus	Ebolavirus	-ssRNA	Reduced titers, reduced protein translation and gene expression	[183]
	Marburgvirus	-ssRNA	Reduced titers	[183]
Rhabdovirus	Rabies virus	-ssRNA	Reduced titers	[89]
	Vesicular stomatitis virus	-ssRNA	Reduced titers	[89]
Retrovirus	Human immunodeficiency virus	RNA/DNA	Reduction in viral production, Rev-dependent nuclear transport, RNA translation and Rev-induced gene expression, transcription initiation	[184,185,186]
Herpesvirus	Herpes simplex virus	dsDNA	Polyamines present in virion to neutralize DNA charge, reduction in DNA synthesis, reduced titers	[158,187,188]
	Human cytomegalovirus	dsDNA	Reduced titers	[159,189]
	Kaposi’s sarcoma-associated herpesvirus	dsDNA	Reduced titers, protein translation, reactivation, and maintenance of latency	[160,161]
Poxvirus	Vaccinia virus	dsDNA	Polyamines present in virion to neutralize DNA charge, reduction in DNA synthesis, reduced titers	[167,190,191,192]

## Data Availability

No new data were created or analyzed in this study.

## Abbreviations

The following abbreviations are used in this manuscript:

ODC1	ornithine decarboxylase 1
ARG1	argininase 1
OAZ1	ODC1 antizyme 1
AZIN	antizyme inhibitor
SAMDC	S-adenosyl methionine decarboxylase
SAT1	spermidine-spermine acetyltransferase 1
DHPS	doxyhypusine synthase
DOHH	deoxyhypusine hydroxylase
eIF5A	eurkaryotic initiation factor 5A
HR	hypersensitive response
SAR	systemic acquired resistance
HCMV	human cytomegalovirus
HSV-1	herpes simplex virus 1
CVB3	Coxsackievirus B3
DFMO	difluoromethylornithine
CAR	Coxsackievirus and adenovirus receptor
SREBP2	sterol response element binding protein 2
AMV	alfalfa mosaic virus
VP30	viral protein 30
KSHV	Kaposi’s sarcoma-associated herpesvirus
PBCV	Paramecium burcella chlorella virus
PSR	polyamine stress response
BCSFB	blood cerebrospinal fluid barrier
TLR3	Toll-like receptor 3
BBB	blood–brain barrier
CNS	central nervous system
PVR	poliovirus receptor
RV	rabies virus
nAchR	nicotinic acetylcholine receptors
ZIKV	Zika virus
CZS	congenital Zika syndrome
NPC	neuronal progenitor cell
WNV	West Nile virus
DENSpm	diethylnorspermine
CHIKV	chikungunya virus
GC7	N1-guanyl-1,7-diamine-heptane
VSV	vesicular stomatitis virus
ASD	autism spectrum disorder
BABS	Bachmann-Bupp syndrome
